# After-Ripening Is Associated with Changes in the Sensitivity of *Avena fatua* L. Caryopses to Abscisic Acid, as Well as Changes in the Abscisic Acid and Bioactive Gibberellins Contents in Embryos

**DOI:** 10.3390/plants14030463

**Published:** 2025-02-05

**Authors:** Jan Kępczyński, Agata Wójcik, Michał Dziurka

**Affiliations:** 1 Institute of Biology, University of Szczecin, Wąska 13, 71-415 Szczecin, Poland; 2 Institute of Plant Physiology, Polish Academy of Sciences, Niezapominajek 21, 20-239 Kraków, Poland

**Keywords:** abscisic acid, caryopsis, dormancy, germination, gibberellins, karrikin

## Abstract

The information on the involvement of hormones in the release of dormancy in grass caryopses is still insufficient. The main aim of the present study was to deepen our understanding of the mechanism dormancy release in *Avena fatua* L. caryopses by after-ripening in relation to abscisic acid (ABA) and gibberellins (GAs). The after-ripening of florets during dry storage resulted in dormancy removal in caryopses, allowing their germination at 20 to 30 °C. Sensitivity to ABA and paclobutrazol (PAC), a GAs biosynthesis inhibitor, decreased as the after-ripening period was extended. After-ripening increased the ABA content in the embryos of dry caryopses and decreased the total content of bioactive GAs, except for GA_1_, the content of which increased. Accordingly, the ABA/GAs ratio was increased, and the ABA/GA_1_ ratio was lowered due to after-ripening. After-ripening led to a decrease in the ABA content and increased the bioactive GAs contents, including GA_1_ and GA_6_, regarded as the most important for germination, in the embryos within 18 h of caryopses germination. The results obtained indicate that the embryos of dormant caryopses contained sufficient levels of bioactive GA_1_ and GA_6_ at early stages of germination, but their germination was prevented due to having too high an ABA concentration. It seems that the agents most important in dormancy removal through after-ripening include a reduction in the ABA content and sensitivity, as well as an increase in the bioactive GAs, particularly GA_1_ and GA_6_, in the embryos of germinating caryopses, which brings about a reduction in the ABA/GAs, ABA/GA_1_, and ABA/GA_6_ ratios, thus enabling germination.

## 1. Introduction

Primary seed dormancy, common mainly in wild plants, prevents the germination of intact viable seeds under conditions (i.e., water, air, temperature) that are favorable for the germination of non-dormant seeds [1]. Depending on the plant species, seed dormancy can be removed by various treatments, e.g., mechanical scarification, chemical scarification, and cold stratification. In many plant species, dormancy can be removed also by after-ripening taking place during the dry storage of seeds. Dormancy release due to dry storage can occur under natural conditions in the field. as well as by dry-storing air-dried seeds in closed containers, in paper bags, or in sealed Petri dishes at room temperature and ambient air humidity [2]. Dry storage results in an increased germination percentage and speed at an optimal temperature, as well as an extension of the temperature range within which germination is possible [3,4]. In general, dormancy release can be sped up by increasing the storage temperature. Studies have shown that dry storage may increase sensitivity to dormancy-releasing factors and decrease vulnerability to seed germination inhibitors [3]. Gibberellins, ethylene, nitric oxide, hydrogen cyanide, and reactive oxygen species have also been used to release dormancy in several plant species [1]. The balance between ABA and GAs and sensitivity to these hormones play a crucial role in the regulation of dormancy and germination. ABA is responsible for the induction of dormancy and its maintenance, whereas GAs participate in dormancy release and seed germination by acting antagonistically to ABA [4].

The florets and caryopses of *Avena fatua*, one of world’s (including Poland) most widespread and persistent annual weeds, are primarily dormant after harvest, with their dormancy being expressed through the lack of germination at warmer temperatures suitable for the germination of non-dormant seeds [5,6]. Physiologically dormant caryopses of *A. fatua* are used as a model system in studies on the mechanism of dormancy release. Dormancy in *A. fatua* caryopses can also be removed by dry storage, as well as treatment with GA_3_, plant-derived smoke, KAR_1_ (identified in plant-derived smoke), and nitric oxide [7,8]. Caryopsis dormancy release by KAR_1_ involves a reduction in caryopsis sensitivity to ABA and the ABA/GAs ratio [9]. In addition, KAR_1_-mediated dormancy release was shown to require ethylene action [10,11]. However, despite numerous studies, the regulation of dormancy release in physiologically dormant seeds by after-ripening needs further investigation [2]. Likewise, there is insufficient information on the effects of various temperatures and durations of storage in *A. fatua* florets on caryopsis germination at various temperatures. It is not known, either, how floret storage at various temperatures affects floret germination. The response of caryopses at different dormancy levels to ABA and to PAC, an inhibitor of gibberellin biosynthesis, is unknown. Moreover, the contents of GAs and ABA/GAs ratios in relation to after-ripening have not been studied.

The present study was aimed at determining: (1) The germination of caryopses and florets following dry storage (after-ripening) at various temperatures for different periods; (2) caryopsis responses to ABA and PAC after various period of dry storage; (3) a relationship between exogenous GA_3_ and ABA, as well as KAR_1_ and ABA in the germination of completely after-ripened (non-dormant) caryopses; (4) the contents of GAs and ABA/GAs ratio in dry embryos from dormant and non-dormant caryopses; (5) the contents of GAs and ABA/GAs ratio in the embryos of germinating dormant and non-dormant caryopses in the absence or the presence of ABA.

The study resulted in new data, allowing for a better understanding of the mechanism, including the consideration of hormone contributions to physiological dormancy release in grass seeds via after-ripening.

## 2. Materials and Methods

*Avena fatua* L. (wild oat) spikelets, containing florets, were collected from wild populations in July 2015 near Szczecin (53.4285° N, 14.5528° E) (Poland). The floret is a single caryopsis covered by lemma and palea. After collection, florets were dried in the open air for 7 days to achieve constant moisture in the caryopses (ca. 11%). In order to maintain primary dormancy, florets were stored at −20 °C until they were needed. Dormancy level did not change during the storage of florets at −20 °C. In order to remove dormancy, primary dormant florets were placed in 1 L jars and dry-stored in darkness at 10, 15, 20, 25, 30, 35, and 40 °C for 2, 4, 8, 12, and 16 weeks. Florets or caryopses (florets without the lemma and palea) were used in the experiments.

### 2.1. Caryopse and Floret Germination After Dry Storage of Florets

Air-dried dormant caryopses (dehulled florets) (25 in 3 replicates) after dry storage of florets at 10, 15, 20, 25, 30, 35, and 40 °C for 2, 4, 8, 12, and 16 weeks were placed in 6 cm diameter Petri dishes on a single layer of filter paper moistened with 1.5 mL distilled water “Table 1 (a,b)”. Petri dishes were kept in darkness at 20, 25, and 30 °C for 7 days. Air-dried dormant florets (25 in 3 replicates) after the dry storage of florets at 20, 25, 30, and 35 °C were placed in 6 cm diameter Petri dishes on a single layer of filter paper moistened with 1.5 mL distilled water. Petri dishes were kept in darkness at 20 °C for 7 days.

### 2.2. Treatment with ABA, PAC, GA_3_, and KAR_1_

After 0, 2, 4, 8, 12 and 16 weeks dry storage of florets at 35 °C caryopses were germinated on distilled water, ABA solutions (10^−5^, 10^−4^ M), or PAC solutions (10^−5^, 10^−4^, 10^−3^ M) at 20 °C for 7 days “Table 1 (c)”. In one experiment, caryopses from florets dry-stored for 16 weeks at 25 °C were germinated on distilled water, GA_3_ (10^−5^, 10^−4^, 3 × 10^−4^ M), KAR_1_ (3 × 10^−9^, 10^−8^ M), ABA (10^−5^, 3 × 10^−5^, 10^−4^ M), GA_3_ + ABA, and KAR_1_ + ABA solutions in the dark at 20 °C for 7 days.

### 2.3. Determination of Germinated Caryopses

**Figure 1:**
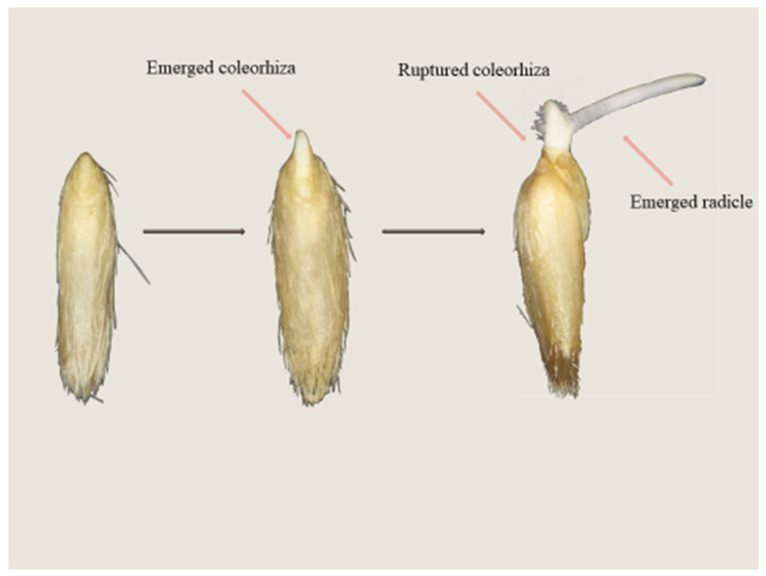
Germination of *A. fatua* caryopses. Germination involves two stages. During the first stage, the coleorhiza breaks through the surrounding structures, and the second stage is associated with radicle emergence. Germination is complete when the coleorhiza is punctured by the radicle.

The caryopses were regarded as germinated when the radicle protruding through the coleorhiza was longer than ca. 1 mm (Figure 1). All manipulations were performed under green light, which did not affect germination. The effects of the compounds used on dormancy release were characterized by the percentage of caryopses germination after 7 days.

### 2.4. Determination of ABA and GAs Contents

Air-dried dormant caryopses and caryopses (25 in 3 replicates) of florets dry-stored at 35 °C for 16 weeks were incubated in the dark at 20 °C in Petri dishes (6 cm diameter) on a single layer of filter paper (Whatman No. 1) moistened with 1.5 mL distilled water for 0, 6, 18, 24, 30, or 36 h. In one experiment, the caryopses from florets dry-stored at 35 °C for 16 weeks were incubated on filter paper moistened with ABA (10^−4^ M) solution for 18 h. Embryos were isolated before and after germination. Data on ABA content in embryos of *A. fatua* dormant and non-dormant dry caryopses or incubated in water and data on the contents of some GAs in the embryos of dormant caryopses incubated in water were derived by the re-analysis of those in published studies [8].

### 2.5. Statistical Treatment

The mean ± standard deviation (SD) of three replicates was calculated. The significance of differences between the means was tested using one- or two-way analysis of variance (ANOVA; Statistica for Windows v. 10.0, Stat-Soft Inc., Tulsa, OK, USA). Duncan’s multiple range test was used to identify significantly different (*p* ≤ 0.05) mean values.

## 3. Results

### 3.1. Effects of Floret Dry Storage at 10–40 °C for Various Periods on Germination of Caryopses at 20, 25, and 30 °C

**Figure 2:**
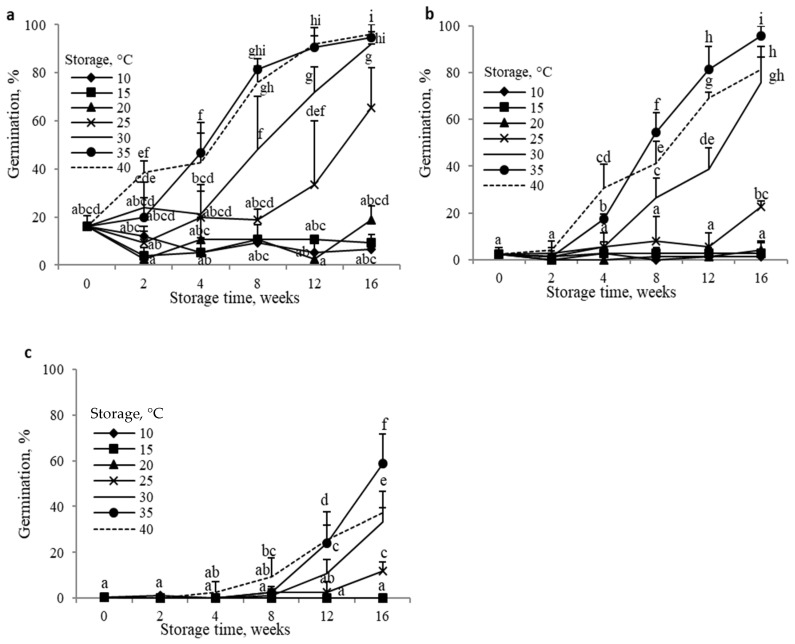
Effects of dry-storing *A. fatua* florets at various temperatures on caryopsis germination at 20 °C (**a**), 25 °C (**b**), and 30 °C (**c**). Vertical bars indicate ±SD. Two-way ANOVA with Duncan’s post hoc test was used to test for significant differences. Means denoted by different letters differ significantly (*p* ≤ 0.05, n = 3).

Caryopses from non-after-ripened (dormant) florets germinated very poorly at 20 °C (Figure 2a) or were unable to germinate at 25 and 30 °C (Figure 2b,c). The dry storage (after-ripening) of florets at 20 °C for up to 16 weeks did not affect caryopsis germination at 20 °C (Figure 2a). A higher storage temperature, 25 °C, increased the germination percentage after 12 and 16 weeks of storage. After 16 weeks, ca. 70% of caryopses were able to germinate. Increasing the storage temperature resulted in increased germination; as many as ca. 95% caryopses germinated when florets were stored for 16 weeks at 35 °C or 40 °C. At 25 °C, caryopses were able to reach the highest germination percentage (80–95%) after florets had been stored for 16 weeks at 35 or 40 °C (Figure 2b). When the temperature of 30 °C was used for germination, the dry storage effect was lower, regardless of the storage temperature (Figure 2c). The highest effect was observed after florets had been stored at 35 °C for 16 weeks; however, only ca. 60% of caryopses were able to germinate.

### 3.2. Effects of Floret Dry Storage at 20–35 °C for Various Periods on Germination of Florets at 20 °C

**Figure 3:**
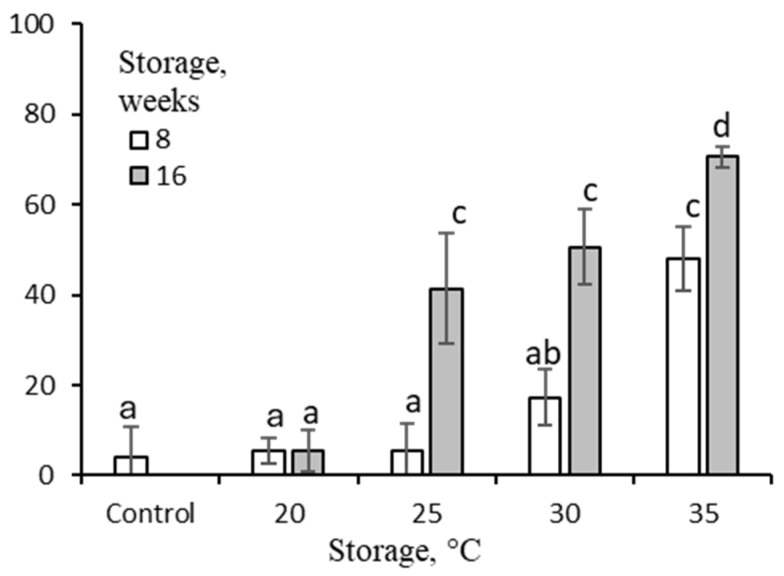
Effects of dry-storing *A. fatua* florets at various temperatures on floret germination at 20 °C. Vertical bars indicate ±SD. Two-way ANOVA with Duncan’s post hoc test was used to test for significant differences. Means denoted by different letters differ significantly (*p* ≤ 0.05, n = 3).

Dormant florets were unable to germinate at 20 °C (Figure 3). Storage for 8 weeks increased the percentage of germination at 20 °C in parallel with the storage temperature. The highest effect (ca. 50%) was recorded when the florets had been stored at 35 °C. The extension of 25–35 °C storage to 16 weeks resulted in increased germination. Most florets (70%) germinated after they had been stored at 35 °C.

### 3.3. Effects of ABA on Caryopsis Germination at 20° C Following Floret Dry Storage at 35 °C for Various Periods

**Figure 4:**
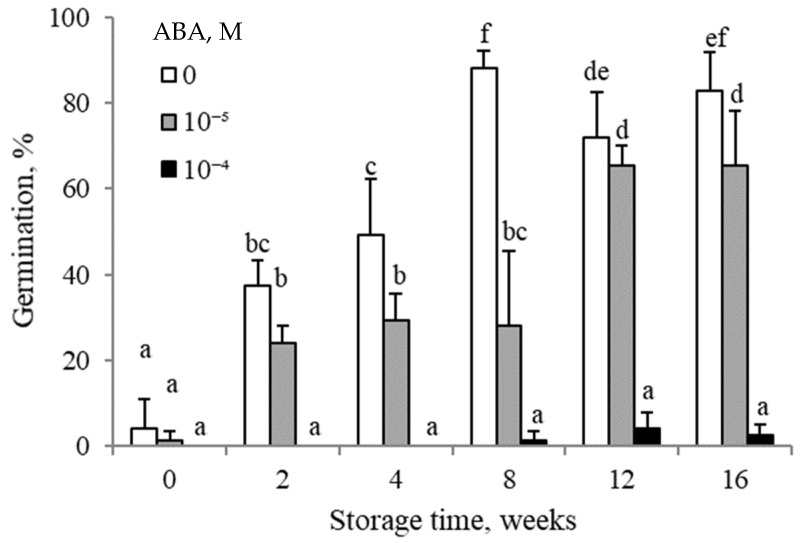
Effects of ABA on germination at 20 °C of *A. fatua* caryopses from florets dry-stored at 35 °C for various periods. Vertical bars indicate ±SD. Two-way ANOVA with Duncan’s post hoc test was used to test for significant differences. Means denoted by different letters differ significantly (*p* ≤ 0.05, n = 3).

To find out whether caryopses with different levels of dormancy differed in response to ABA, the effects of the compound on caryopsis germination after various periods of floret dry storage at 35 °C were examined (Figure 4).

ABA was found to inhibit germination, with the inhibition level being related to the concentration and storage time. ABA applied at 10^−5^ M inhibited caryopsis germination after 4 and 8 weeks of floret dry storage. However, the inhibitory effect of ABA decreased when florets had been stored for 12 and 16 weeks. When applied at a higher concentration (10^−4^ M), ABA inhibited germination completely, regardless of the floret dry storage duration.

### 3.4. Effects of PAC on Caryopsis Germination at 20 °C Following Floret Dry Storage at 35 °C for Various Periods

**Figure 5:**
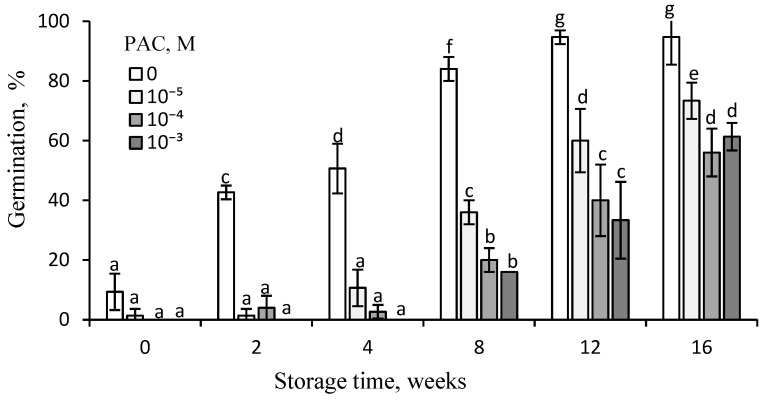
Effects of PAC on the germination at 20 °C of *A. fatua* caryopses from florets dry-stored at 35 °C for various periods. Vertical bars indicate ±SD. Two-way ANOVA with Duncan’s post hoc test was used to test for significant differences. Means denoted by different letters differ significantly (*p* ≤ 0.05, n = 3).

PAC, a gibberellin biosynthesis inhibitor, was found to inhibit caryopsis germination, with the effect being dependent on the PAC concentration and the duration of storage (Figure 5).

The inhibitory effect decreased with the extension of the storage duration and was at its lowest after 12 and 16 weeks of storage. After 4 weeks of storage, ca. 50% of caryopses germinated in water, but almost none germinated in the presence of PAC, regardless of concentration. After the florets had been stored for 12 weeks, a high level of inhibition by PAC was still evident. PAC applied at 10^−5^ M almost halved the caryopsis germination percentage; about one third of caryopses germinated with PAC applied at concentrations of 10^−4^–10^−3^ M. After 16 weeks of floret storage, ca. 70 or 60% of caryopses germinated at 10^−5^ or 10^−4^–10^−3^ M PAC, respectively, whereas 95% of caryopses from dry-stored florets germinated in the absence of PAC.

### 3.5. Effects of GA_3_ on Caryopsis Germination at 20 °C in the Absence or in the Presence of ABA Following Floret Dry Storage at 25 °C for 16 Weeks

**Figure 6:**
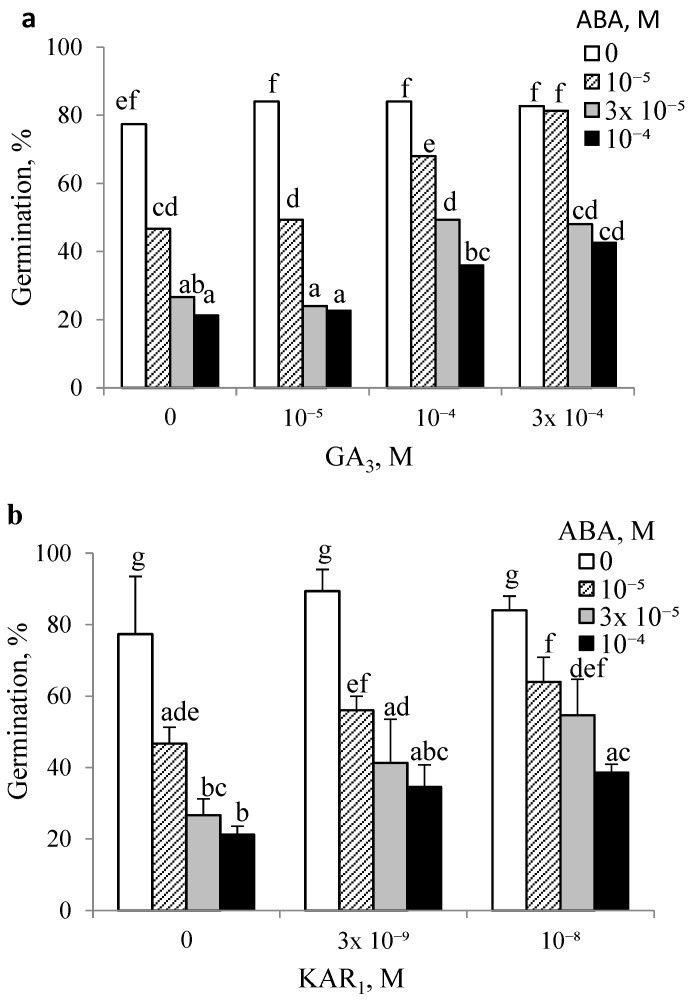
Effects of GA_3_ (**a**) and KAR_1_ (**b**) on germination at 20 °C in the absence or in the presence of ABA of non-dormant *A. fatua* caryopses. Non-dormant caryopses were obtained after floret dry storage at 25 °C for 16 weeks. Vertical bars indicate ±SD. Two-way ANOVA with Duncan’s post hoc test was used to test for significant differences. Means denoted by different letters differ significantly (*p* ≤ 0.05, n = 3).

GA_3_ at all concentrations did not affect the germination percentage of non-dormant (after-ripened for 16 weeks) caryopses in the absence of ABA (Figure 6a). GA_3_ used at 10^−4^ and 3 × 10^−4^ M almost doubled the germination percentage, despite the presence of 3 × 10^−5^ or 10^−4^ M ABA; ca. 40% of caryopses germinated in the presence of both compounds and ca. 20% germinated when ABA alone was applied.

### 3.6. Effect of KAR_1_ on Caryopsis Germination at 20 °C in the Absence or the Presence of ABA Following Floret Dry Storage at 25 °C for 16 Weeks

In contrast to ABA, which markedly inhibited the germination of non-dormant caryopses, KAR_1_ at concentrations of 3 × 10^−9^ or 10^−8^ M did not produce any significant effect (Figure 6b). Germination was increased when KAR_1_ was applied in the presence of various concentrations of ABA. The antagonizing effect of KAR_1_ was higher when its higher concentrations were applied. KAR_1_ used at 10^−8^ M doubled the germination percentage in the presence of 10^−4^ M ABA; ca. 40% of caryopses were able to germinate.

### 3.7. ABA and GAs Contents and ABA/GAs Ratios in Embryos from Dormant and Non-Dormant Dry Caryopses

Bioactive gibberellins from both the 13-hydroxylation (GA_1_, GA_5_, GA_3_, GA_6_) and non-13-hydroxylation (GA_4_, GA_7_) pathways were detected in the embryos of dry dormant and non-dormant caryopses (Table 2). Moreover, GA_20_ and GA_9_, precursors of GA biosynthesis in 13-hydroxylation and non-13-hydroxylation, respectively, were identified, as well. To find out whether after-ripening resulting in increased germination capacity involved GAs, the contents of various GAs in the embryos of dry dormant and dry-stored caryopses were compared. The contents of GA_5_, GA_6_, and GA_4_ decreased as a result of dry storage. However, only GA_1_ increased by ca. 70% due to after-ripening. The total bioactive GAs content in non-dormant embryos was lower than that in dormant ones by ca. 40% (Table 3 and Table 4). The high initial ABA content in dry dormant embryos increased by ca. 20% due to the dry after-ripening of florets (Table 3 and Table 4). The ABA/GAs ratio in non-dormant embryos was two times higher than in dormant embryos. However, the ABA/GA_1_ ratio in non-dormant embryos was 1.4 times lower than in dormant embryos.

### 3.8. ABA and GAs Contents and ABA/GAs Ratios in Embryos from Dormant and Non-Dormant 18 h Germinating Caryopses

To find out the role of ABA and GAs in dormancy, the contents of both hormones in the embryos of dormant and after-ripened caryopses after germination for 18 h were also analyzed. The contents of all GAs from the 13-hydroxylation pathway in dormant (except GA_6_) and non-dormant embryos germinating for 18h were higher than those in dry dormant and non-dormant embryos (Table 2). The contents of all GAs from the non-13-hydroxylation pathway, GA_4_ and GA_7_, increased after 18 h germination when florets had previously been after-ripened. The total contents of all bioactive GAs in the embryos of germinating dormant and non-dormant caryopses were similar (Table 3 and Table 4); the GAs content increased 1.4 and 2.2 times during 18 h germination in dormant and after-ripened caryopses, respectively. Dormant and non-dormant embryos from germinating caryopses showed the highest GA_1_ content compared to other GAs from both pathways; GA_1_ accounted for almost 50% of the total bioactive GAs. Of all the GAs, the GA_6_ content was second only to that of GA_1_. GA_1_ also presented at high concentrations during the incubation of dormant caryopses for 36 h (Table 5). The ABA content increased by 20% in dormant embryos when caryopses were germinated for 18 h (Table 3). The ABA content doubled in after-ripened embryos after the germination of caryopses for 18 h (Table 4). The ABA/GAs and ABA/GA_1_ ratios in non-dormant embryos were lower than those in dormant ones by a factor of ca. 1.9 (Table 3 and Table 4).

### 3.9. Effects of ABA on ABA and GAs Contents and ABA/GAs Ratio in Embryos from Caryopses Germinating for 18 h, Obtained from Florets Previously Dry-Stored at 35 °C for 16 Weeks

**Figure 7:**
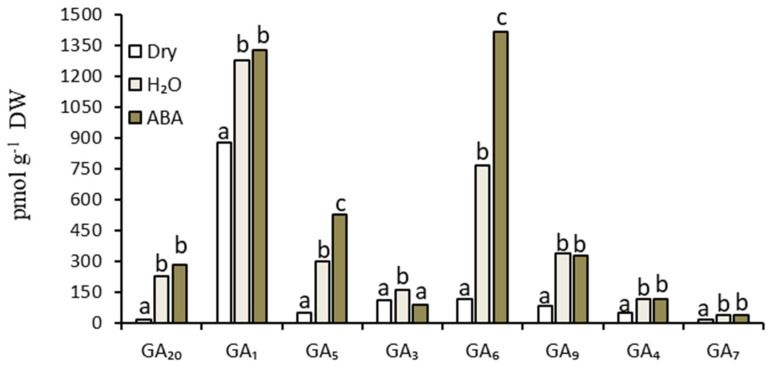
Effects of ABA on contents of different GAs in the embryos of germinating non-dormant *A. fatua* caryopses. Non-dormant caryopses were obtained after floret dry storage at 35 °C for 16 weeks. Caryopses were germinated at 20 °C for 18 h. Two-way ANOVA with Duncan’s post hoc test was used to test for significant differences. Means denoted by different letters differ significantly (*p* ≤ 0.05, n = 3).

The germination of non-dormant caryopses in the presence of ABA for 18 h greatly increased (up to about 70-fold) the ABA content in embryos (Table 4). ABA present over 18 h of germination in non-dormant caryopses increased the total bioactive GA content in embryos by ca. 30%, with the GA_5_ and GA_6_ contents increasing by ca. 80–90% (Table 4, Figure 7).

When caryopses were germinated in the presence of ABA, the ABA/GA and ABA/GA_1_ ratios increased greatly (by a factor of ca. 50–60) compared to the caryopses germinated in water (Table 4).

## 4. Discussion

### 4.1. Responses of Caryopses and Florets to Floret After-Ripening

The caryopses of *A. fatua* (wild oat)*,* a common monocot weed, can germinate only at very a low level, usually 5 to 20%, at 20 °C, and are not able to germinate at 30 and 35 °C (Figure 2 and Figure 4). However, they are able to germinate at lower temperatures; e.g., at 10 °C, the germination percentage is ca. 40% [12]. Therefore, like in temperature cereals [4], the expression of dormancy in *A. fatua* caryopses depends on the incubation temperature used during germination. Like in the seeds of many species of weeds and cultivated plants [2,3], dormancy in *A. fatua* caryopses can be removed by after-ripening during dry storage [8,13] (Figure 2). For example, seed dormancy in the dicot *Amaranthus retroflexus*, also an important common weed, can be released as a result of dry storage for 5 months at 24–28 °C [14] or for 16 weeks at 35 °C, allowing for ca. 70% germination [15]. Dormancy in *A. fatua* caryopses was released by dry-storing the florets for 6 months at 30 or 40 °C [16] or for 12 weeks at 25 °C [10], whereby almost all the caryopses were observed to be germinating. It was previously recommended that studies investigating dormancy release via after-ripening should test germination at different temperatures of dormant seeds and seeds after-ripened at different temperatures for different periods of time [2]. Accordingly, we tested the effects of five storage temperatures for *A. fatua* florets on caryopsis germination at 20, 25, and 30 °C in order to characterize the dormancy level in response to storage conditions. At 20 °C, non-stored dormant caryopses were almost unable to germinate (Figure 2a and Figure 4). However, dry storage at 25 to 40 °C removed dormancy, which resulted in a high germination percentage. As with many other seeds [2], the rate of dormancy release increased with increasing storage temperature; the greatest effect was observed at 35 or 40 °C (Figure 2). The effectiveness of dry storage was found to be dependent on the temperature during germination, with the effect being less evident at higher germination temperatures. Thus, dormancy is deeper at higher germination temperatures than at lower germination temperatures. The dry storage of florets increases germination in caryopses more effectively than in florets, suggesting a deeper dormancy in florets than in caryopses (Figure 2 and Figure 3). The higher dormancy level in florets than in caryopses may be related to the presence of certain structures, the lemma and the palea, outside the caryopsis [5]. On the other hand, the coleorhiza plays a major role in caryopsis dormancy and germination in *A. fatua* [13,17] and *Hordeum vulgare* [18], acting as a barrier to radicle emergence.

### 4.2. Caryopsis Response to ABA and PAC Following Floret After-Ripening

The response of caryopses to exogenous ABA turned out to be lower if the florets had been after-ripened for a longer time (Figure 4). This agrees with the results of experiments involving seeds of, e.g., *Triticum aestivum*, *H. annuus*, and *A. retroflexus*, which showed a decreased responsiveness to ABA as dry after-ripening was extended [15,19,20]. The ABA content in embryos at an early stage of their germination was shown to be reduced by the dormancy-releasing KAR_1_ and also by caryopsis after-ripening [13] (Table 4). Likewise, earlier studies demonstrated that the ABA level in barley embryos was decreased by after-ripening [18,21].

PAC, commonly regarded as an inhibitor of gibberellin biosynthesis [22], was also applied to caryopses after various periods of after-ripening to assess the magnitude of caryopsis response in relation to the level of dormancy, to assess the contribution of GAs synthesis to dormancy release. Both the after-ripened caryopses (Figure 5) and *H. annuus* seeds [20] were able to respond to PAC. The caryopsis responsiveness to PAC was revealed to decrease with the extension of the dry storage duration, suggesting that dormancy release due to after-ripening was associated with increasing GAs contents. This is also in agreement with previous findings that dormancy release in *A. fatua* caryopses by KAR_1_ involves an increase in the GAs content in embryos and radicles [9]. Moreover, dormancy can be removed by GA_3_ and KAR_1_, and only the KAR_1_ effect was strongly counteracted by PAC [12], which reduced the GAs content in the embryos of KAR_1_-treated caryopses [9]. Thus, it may be assumed that a decreasing response to PAC with increasing storage time is rather related to an increased ability to synthesize GAs. It seems important to point out that PAC can also perform another function because, when used alone or simultaneously with KAR_1_, it increases the ABA content in *A. fatua* embryos [9].

In view of the data obtained, it can be concluded that dormancy release due to after-ripening may involve a decrease in responsiveness towards ABA and possibly an increase in GAs synthesis.

### 4.3. Relationship Between Exogenous ABA and GA_3_ or KAR_1_ in Relation to Germination of Non-Dormant Caryopses

GA_3_ and KAR_1_, in contrast with ABA, did not affect the germination of completely after-ripened caryopses (Figure 6). Like in previous experiments with dormant caryopses [9], GA_3_ and KAR_1_ were able to antagonize the inhibitory effect of ABA on the percentage of germination in non-dormant caryopses (Figure 6). In view of the percentage of germination, GA_3_ may be concluded to be less effective than KAR_1_ in antagonizing ABA inhibition, similarly to the findings of previous studies [9]. Likewise, experiments with other dormant seeds, e.g., *Arabidopsis thaliana* and *Brassica tournefortii* [23,24,25], demonstrated a lower activity of GA_3_ than KAR_1_.

### 4.4. ABA and GAs Contents in Embryos of Dry Caryopses in Relation to After-Ripening

Dormancy and germination are regarded as being controlled primarily by the balance between ABA and GAs. While ABA is considered to be responsible for dormancy induction and maintenance, as well as germination inhibition, GAs function as an ABA antagonist and germination inducer [26,27]. The need for GAs to release dormancy and induce germination in *Arabidopsis* seeds is determined by the ABA produced during seed development and/or imbibition [27]. It has been postulated that GAs act first, stimulating *Arabidopsis* seed germination before ABA levels decline, and ABA acts as the final checkpoint preventing germination [28].

The role of ABA and GAs in dormancy release can be studied in two ways. The contents of the hormones can be compared in dry dormant seeds and in dry seeds after dry storage. Alternatively, the levels of ABA and GAs can be compared in germinating dormant and after-ripened seeds. A comparison of the ABA contents in embryos from dry dormant caryopses of *A. fatua* and dry caryopses of after-ripened florets showed that storage resulted in an increased ABA content in embryos [13] (Table 3 and Table 4). This is consistent with early studies showing that the ABA content in embryos from after-ripened *A. sativa* caryopses was higher than that in dormant embryos [29]. Likewise, an increase in the ABA level in dry *Arabidopsis* seeds via after-ripening was demonstrated, as well [28]. To check whether after-ripening, which causes an increase in the germination capacity of *A. fatua*, involves biologically active GAs, their contents in dry dormant and non-dormant embryos were determined. Like in seeds of many other species [27], GAs from two pathways were identified (Table 2). GA_1_ and GA_3_ from the 13-hydroxylation pathway, as well as GA_4_ and GA_7_ from the non-hydroxylation pathway, commonly recognized as major bioactive GAs [30], were found to be present in both dry dormant and dry non-dormant embryos (Table 2). Moreover, the presence of bioactive GA_5_ and GA_6_, as well as GA_20_ and GA_9_, precursors of GAs biosynthesis in the 13-hydroxylation and non-hydroxylation pathways, respectively, were found, as well. After-ripening brought about a reduction in the content of three bioactive gibberellins and the total GAs content in embryos of dry caryopses (Table 2 and Table 4). It is worth emphasizing that the content of GA_1_, regarded as the main bioactive GA in wheat [31,32], increased by about 70% as a result of after-ripening. Moreover, it seems important that GA_1_ accounted for about 70% of all GAs. It can be assumed that it was formed, during after-ripening, from GA_20_ present in the embryos of dry dormant caryopses. The ABA/GAs ratio in dry embryos was shown to increase by a factor of 1.9 due to after-ripening (Table 3 and Table 4). However, the ABA/GA_1_ ratio decreased by a factor of 1.4 due to the increased content of GA_1_. Considering that GA_1_ seems to be the most important GA, since it occurs at the highest concentration, the ABA/GA_1_ ratio best characterizes the effectiveness of after-ripening. To sum up, despite the increased ABA content, the after-ripening-induced dormancy release in caryopses also resulted in an increased content of GA_1_, which was the cause of the decreased ABA/GA_1_ ratio. Therefore, the effect of after-ripening on the dormancy release in *A. fatua* is associated with a decrease in the ABA/GA_1_ ratio.

### 4.5. ABA and GAs Contents in Embryos of Germinating Caryopses in Relation to After-Ripening

The role of ABA and GAs in releasing dormancy by after-ripening was also assessed by analyzing the contents of these hormones in embryos during early stage of germination (after 18 h) in dormant and non-dormant *A. fatua* caryopses. The increased level of ABA observed during the germination of dormant caryopses (Table 3) and sunflower seeds [33] confirms the important role of ABA in the maintenance of dormancy. Like in barley embryos [34] and *Brachypodium distachyon* grains [35], as well as sunflower seeds [33], the ABA level in embryos of germinating *A. fatua* caryopses [13] (Table 3 and Table 4) was reduced by after-ripening. The total GAs level, as well as that of GA_1_ at the same caryopses germination time, was fairly similar in dormant and non-dormant embryos (Table 2, Table 3 and Table 4). However, after-ripening increased the total GAs and GA_1_ contents in the embryos of germinating caryopses compared to the content in embryos from dry after-ripened caryopses, by a factor of 2.2 and 1.5, respectively. It should be emphasized that GA_1_ accounted for about 50% of the total bioactive GAs content in the embryos of germinating after-ripened caryopses. Thus, these findings may be taken as confirmation of GA_1_ playing the most important role in caryopsis dormancy release and germination. The contribution of GAs to dormancy removal in caryopses was also demonstrated in previous studies. Namely, KAR_1_-induced dormancy release in *A. fatua* caryopses was found to be associated with an increase in the content of bioactive GAs in the embryos of germinating caryopses [9]. Moreover, the KAR_1_ effect involves an increased GAs content, particularly that of GA_1_, in the radicle. There are findings showing that dormancy release due to after-ripening is associated with an increased GAs content in the embryos of germinating sunflowers [33] and *Arabidopsis* seeds [28], as well as with an increased GA_1_ content in wheat [31].

It seems that, in addition to elucidating the role of GA_1_, the importance of GA_6_ is also worthy of attention, with GA_6_ assumed to be the second most important GA involved in the control of dormancy and germination in *A. fatua* caryopses. GA_6_, considered a stable GA that may serve for transport or accumulation [27], accounted for 30% of the total GAs content (Table 2 and Table 4). Likewise, previous studies demonstrated that the release of *A. fatua* caryopsis dormancy by KAR_1_ was associated with a significant elevation in GA_6_ content in embryos at an early stage of caryopsis germination (18 h), and at the late stage (36 h) prior to radicle emergence, when ca. 50% of the coleorhiza emerged [9]. Furthermore, the GA_6_ content was also significantly elevated in the radicle prior to its emergence [9]. In addition, GA_6_ has previously been proposed as an important GA involved in the germination of dicot *Lepidium sativum* seeds [36]. Interestingly, the total GAs content in embryos of germinating after-ripened caryopses was almost identical to that in embryos of germinating dormant caryopses (Table 3 and Table 4). However, dormant caryopses did not germinate, despite the GAs contents (especially those of GA_1_ and GA_6_) being sufficient for dormancy release and germination (Table 2) and GA_1_ being sufficient even up to 36 h of germination (Table 5). This can likely be explained by the too-high ABA content. The ABA/GA_1_ and ABA/GA_6_ ratios in the embryos of germinating non-dormant caryopses were shown to be reduced by after-ripening, due to both a decrease in the ABA contents and an increase in these GAs (Table 2 and Table 4), which points to the most important role of the ABA/GAs ratios in controlling the state of dormancy. Likewise, dormancy loss in wheat seeds has been shown to be associated with the regulation of the ABA/GAs ratio [32].

### 4.6. ABA and GAs Contents in Embryos of Germinating Non-Dormant Caryopses in Relation to Exogenous ABA

Exogenous ABA more effectively inhibited the second stage of germination in non-dormant caryopses, i.e., radicle emergence [13] (Figure 6), than the first one, i.e., coleorhiza emergence [13]. The inhibition of germination (Figure 6) was associated with a huge increase in the ABA content (Table 4). Surprisingly, exogenous ABA increased the total GAs, mainly GA_5_ and GA_6_ (Table 2 and Table 4, Figure 7). This could be interpreted as a side effect of ABA being present at a high concentration. ABA was also able to increase gibberellin-like substances and GA_4_ in the leaves of *Solanum andigena* [37] and *Cucumis melo*, respectively [38]. It can be assumed that the inhibition of germination by ABA is associated with the extremely high ABA content in embryos as the cause of a huge increase in the ABA/GAs ratio, despite the increase in the GAs contents. This also supports the notion that ABA is of crucial importance as a factor responsible for blocking the germination of dormant caryopses.

## 5. Conclusions

Dormancy in *A. fatua* florets and caryopses can be released during the after-ripening of florets, with the highest effect being obtained at a storage temperature exceeding 20 °C. Florets are more dormant than caryopses; therefore, the dry storage of florets at various temperatures was less effective in releasing their dormancy than caryopses dormancy. Caryopsis dormancy expression is more visible at germination temperatures exceeding 20 °C; thus, after-ripening is less effective at these temperatures.

After-ripening-associated dormancy removal was related to a decreasing sensitivity to ABA and the GAs synthesis inhibitor. Both GA_3_ and KAR_1_ were able to antagonize the inhibitory effect of exogenous ABA on germination in non-dormant caryopses; however, KAR_1_ proved to be more effective. This antagonistic interaction between GA_3_ and ABA suggests a similar interaction between endogenous GAs and ABA. The ABA contents increased, but those of GAs decreased in dry embryos due to after-ripening, leading to an increase in the ABA/GAs ratio. However, the GA_1_ content increased markedly in dry embryos due to after-ripening, considerably reducing the ABA/GA_1_ ratio. It appears that after-ripening induces a very important change in embryos at the early stage of caryopsis germination (18 h), enabling emergence from dormancy and germination. Namely, after-ripening led to a decrease in ABA content and an increase in the contents of GAs (particularly GA_1_ and GA_6_, which are regarded as the most important for germination) in the embryos of germinating caryopses, which was the cause of the decrease in the total ABA/GAs, ABA/GA_1_, and ABA/GA_6_ ratios.

ABA produced during *A. fatua* caryopsis development but only partly produced during the initial stage of germination is mainly responsible for dormancy in caryopses. It seems that ABA plays a key role in maintaining caryopsis dormancy at early stages of germination (18 h) by preventing GAs, particularly GA_1_ and GA_6_, from fulfilling their function, despite their sufficient contents. Moreover, the assessment of the influence of exogenous ABA on the huge increase in its content and the increased ABA/GAs and ABA/GA_1_ ratios in embryos of germinating caryopses, despite the increased GAs content, as well as the inhibition of germination, may be taken as a confirmation of the essential function of ABA in maintaining dormancy.

The mechanism behind caryopsis dormancy release by after-ripening is associated with a decreased sensitivity to ABA, as well as a decrease in ABA content and an increase in bioactive GAs contents causing a reduction in ABA/GAs, ABA/GA_1_, and ABA/GA_6_ ratios in the embryos of germinating caryopses.

## Figures and Tables

**Table 1:** Scheme of experiments described in Section 2.1 and Section 2.2.

Florets Dry Storage (After-Ripening)		Germination	
Temperature, °C	Time, Weeks	Florets/Caryopses	Temperature, °C
a. 10, 15, 20, 25, 35, 40	2, 4, 8, 12, 16	Caryopses	20, 25, 30 (H_2_ O)
b. 20, 25, 30, 35	8, 16	Florets	20 (H_2_ O)
c. 35	2, 4, 8, 12, 16	Caryopses	20 (H_2_ O, ABA or PAC)

**Table 2:** Contents of different GAs in the embryos of dry and germinating dormant and non-dormant *A. fatua* caryopses. Non-dormant caryopses were obtained after floret dry storage at 35 °C for 16 weeks. Caryopses were germinated at 20 °C for 18 h. The contents of GAs are expressed in pmol/g^−1^ DW. One-way ANOVA with Duncan’s post hoc test was used to test for significant differences. Means denoted by different letters differ significantly (*p* ≤ 0.05, n = 3).

Gibberellins	Dormant		Non-Dormant	
	Time, h			
	0	18	0	18
GA_20_	81.5 ± 24 b	166.0 ± 36 c	15.6 ± 4 a	253.6 ± 18 d
GA_1_	519.8 ± 97 a	1356.0 ± 43 c	880.0 ± 21 b	1279.0 ± 116 c
GA_8_	59.8 ± 11 c	159.4 ± 11 d	26.7 ± 3 b	7.0 ± 0.5 a
GA_5_	156.2 ± 14 b	358.7 ± 56 c	52.0 ± 5 a	300.0 ± 20 c
GA_3_	83.4 ± 10 a	220.8 ± 54 c	114.7 ± 23 ab	163.0 ± 21 bc
GA_6_	790.1 ± 125 b	749.8 ± 77 b	117.7 ± 24 a	772.7 ± 68 b
GA_9_	196.9 ± 27 b	388.6 ± 18 d	86.0 ± 20	313.0 ± 21 c
GA_4_	416.7 ± 46 c	162.2 ± 45 b	49.0 ± 5 a	124.7 ± 20 b
GA_7_	19.9 ± 3 a	38.7 ± 4 b	20.0 ± 5 a	39.3 ± 2 b

**Table 3:** Contents of ABA and total bioactive GAs and ABA/GA_1_ and ABA/GAs ratios in embryos from dry or germinating dormant caryopses of *A. fatua*. Caryopses were germinated at 20 °C for 18 h. One-way ANOVA with Duncan’s post hoc test was used to test for significance of differences. Means denoted by different letters differ significantly (*p* ≤ 0.05, n = 3).

Treatment	ABA	GAs	ABA/GA_1_	ABA/GAs
	nmol/g^−1^ DW			
0	2.02 ± 0.15 a	1.99 ± 0.06 a	3.89	1.02
H_2_O	2.43 ± 0.11 b	2.88 0.07 b	1.84	0.84

**Table 4:** Effects of ABA on the contents of ABA and total bioactive GAs and ABA/GA_1_ and ABA/GAs ratios in embryos from dry or germinating non-dormant *A. fatua* caryopses. Non-dormant caryopses were obtained after floret dry storage at 35 °C for 16 weeks. Caryopses were germinated at 20 °C for 18 h. One-way ANOVA with Duncan’s post hoc test was used to test for significant differences. Means denoted by different letters differ significantly (*p* ≤ 0.05, n = 3).

Treatment	ABA	GAs	ABA/GA_1_	ABA/GAs
	nmol/g^−1^ DW			
0	2.40 ± 0.18 b	1.23 ± 0.04 a	2.72 ± 0.2	1.95
H_2_O	1.21 ± 0.01 a	2.70 ± 0.14 b	0.96 ± 0.1	0.45
ABA	82.50 ± 3.14	3.53 ± 0.23 c	61.0 ± 8.7	23.37

**Table 5:** Changes in GA_1_ content and ABA/GA_1_ ratio in embryos during 36 h germination in dormant *A. fatua* caryopses. One-way ANOVA with Duncan’s post hoc test was used to test for significant differences. Means denoted by different letters differ significantly (*p* ≤ 0.05, n = 3).

Germination Time, h	GA_1,_ pmol/g^−1^ DW	ABA/GA_1_
6	564.3 ± 96.8 a	4.3
24	1536.1 ± 88.2 c	1.5
30	1928.8 ± 48.4 d	1.1
36	1230.0 ± 137.2 b	1.3

## Data Availability

Data are contained within the article.

## References

[1] Bewley J.D., Bradford K.J., Hilhorst H.W.M., Nonogaki H., Bewley J.D., Bradford K.J., Hilhorst H.W.M., Nonogaki H. (2013). Germination. Seeds: Physiology of Development, Germination and Dormancy.

[2] Baskin C.C., Baskin J.M. (2020). Breaking seed dormancy during dry storage: A useful tool or major problem for successful restoration via direct seeding?. Plants.

[3] Iglesias-Fernandez R., Rodríguez-Gacio M.C., Matilla A.J. (2011). Progress in research on dry after-ripening. Seed Sci. Res..

[4] Rodríguez M.V., Barrero J.M., Corbineau F., Gubler F., Benech-Arnold R.L. (2015). Dormancy in cereals (not too much, not so little): About the mechanisms behind this trait. Seed Sci. Res..

[5] Simpson G.M. (1990). Seed Dormancy in Grasses.

[6] Kępczyński J., Mukherjee S., Aftab T. (2023). Induction of dormancy release in agricultural weed seeds by plant-derived smoke and smoke-derived Karrikin1 (KAR1). A relationship with plant hormones. Strigolactones, Karrikins and Alkamides in Plants.

[7] Kępczyński J. (2018). Induction of agricultural weed seed germination by smoke and smoke-derived karrikin (KAR_1_), with a particular reference to *Avena fatua* L.. Acta Physiol. Plant..

[8] Kępczyński J., Wójcik A., Dziurka M. (2023). NO-mediated dormancy release of *Avena fatua* caryopses is associated with decrease in abscisic acid sensitivity, content and ABA/GA_s_ ratios. Planta.

[9] Kępczyński J., Dziurka M., Wójcik A. (2024). KAR_1_-induced dormancy release in *Avena fatua* caryopses involves reduction of caryopsis sensitivity to ABA and ABA/GA_s_ ratio in coleorhiza and radicle. Planta.

[10] Kępczyński J., Van Staden J. (2012). Interaction of karrikinolide and ethylene in controlling germination of dormant *Avena fatua* L. caryopses. Plant Growth Regul..

[11] Ruduś I., Cembrowska D., Jaworska A., Kępczyński J. (2019). Involvement of ethylene biosynthesis and perception during germination of dormant *Avena fatua* L. caryopses induced by KAR_1_ or GA_3_. Planta.

[12] Ruduś I., Kępczyński J. (2017). Exogenous putrescine increases the responsiveness of thermodormant *Avena fatua* L.caryopses to karrikinolide and gibberellic acid. Acta Physiol. Plant..

[13] Kępczyński J., Wójcik A., Dziurka M. (2021). *Avena fatua* caryopsis dormancy release is associated with changes in KAR_1_ and ABA sensitivity as well as with ABA reduction in coleorhiza and radicle. Planta.

[14] Schonbeck M.W., Egley G.H. (1980). Redroot pigweed (*Amaranthus retroflexus*) seed germination responses to after-ripening, temperature, ethylene and some other environmental factors. Weed Sci..

[15] Kępczyński J., Sznigir P. (2014). Participation of GA_3_, ethylene, NO and HCN in germination of L. seeds with various dormancy levels. Acta Physiol. Plant..

[16] Foley M.E. (1994). Temperature and water status of seed after-ripening in wile oat (*Avena fatua*). Weed Sci..

[17] Holloway T., Steinbrecher T., Perez M., Seville A., Stock D., Nakabashi K., Leubner-Metzger G. (2020). Coleorhiza-enforced seed dormancy: A novel mechanism to control germination in grasses. New Phytol..

[18] Barrero J.M., Talbot M.J., White R.G., Jacobsen J.V., Gubler F. (2009). Anatomical and transcriptomic studies of the coleorhiza reveal the importance of this tissue in regulating dormancy in barley. Plant Physiol..

[19] Tuttle K.M., Martinez S.A., Schramm E.C., Takebayashi Y., Seo M., Steber C.M. (2015). Grain dormancy loss is associated with changes in ABA and GA sensitivity and hormone accumulation in bread wheat, *Triticum aestivum* (L.). Seed Sci. Res..

[20] Rodríguez M.V., Bodrone M.P., Castellari M.P., Batilla D. (2018). Effect of storage temperature on dormancy release of sunflower (*Helianthus annuus*) achenes. Seed Sci. Res..

[21] Gubler F., Hughes T., Waterhouse P., Jacobsen J. (2008). Regulation of dormancy in barley by blue light and after-ripening: Effects on abscisic acid and gibberellin metabolism. Plant Physiol..

[22] Desta B., Amare G. (2021). Paclobutrazol as a plant growth regulator. Chem. Biol. Technol. Agric..

[23] Daws M.I., Davies J., Pritchard H.W., Brown N.A.C., Van Staden J. (2007). Butenolide from plant-derived smoke enhances germination and seedling growth of arable weed species. Plant Growth Regul..

[24] Stevens J.C., Merritt D.J., Flematti G.R., Ghisalberti E.L., Dixon K.W. (2007). Seed germination of agricultural weeds is promoted by the butenolide 3-methyl-2H-furo[2,3-c]pyran-2-one under laboratory and field conditions. Plant Soil.

[25] Nelson D.C., Riseborough J.A., Flematti G.R., Stevens J., Ghisalberti E.L., Dixon K.W., Smith S.M. (2009). Karrikins discovered in smoke trigger *Arabidopsis* seed germination by a mechanism requiring gibberellic acid synthesis and light. Plant Physiol..

[26] Finch-Savage W.E., Leubner-Metzger G. (2006). Seed dormancy and the control of germination. New Phytol..

[27] Urbanova T., Leubner-Metzger G. (2016). Gibberellins and seed germination. Ann. Plant Rev..

[28] Nelson K., Kanno Y., Sepo M., Steber C.M. (2023). Seed dormancy loss from dry after-ripening is associated with increasing gibberellin hormone levels in *Arabidopsis thaliana*. Front. Plant Sci..

[29] Poljakoff-Mayber A., Popilevski I., Belausov E., Ben-Tal Y. (2002). Ivolvement of phytohormones in germination of dormant and non-dormant oat (*Avena sativa* L.). Plant Growth Regul..

[30] Yamaguchi S. (2008). Gibberellins metabolism and its regulation. Ann. Rev. Biol..

[31] Kashiwakura Y.-I., Jikumaru Y., Kobayashi D., Takebayashi Y., Nambara E., Seo M., Kamiya Y., Kushiro T., Kawakami N. (2016). Highly sprouting-tolerant wheat grain exhibits extreme dormancy and cold imbibition-resistant accumulation of abscisic acid. Plant Cell Physiol..

[32] Tuan P.A., Kumar R., Rehal P.K., Toora P.K., Ayele B.T. (2018). Molecular mechanism underlying abscisic acid/gibberellin balance in the control of seed dormancy and germination in cereals. Front. Plant Sci..

[33] Xia Q., Ponnaiah M., Thanikathansubramanian K., Corbineau F., Bailly C., Nambara E., Meimoun P., El-Maarouf-Bouteau H. (2019). Re-localization of hormone effectors is associated by temperature and after-ripening in sunflower seeds. Sci. Rep..

[34] Jacobsen J.V., Pearce D.W., Poole A.T., Pharis R.P., Mander L.N. (2002). Abscisic acid, phaseic acid and gibberellin contents associated with dormancy and germination in barley. Physiol. Plant..

[35] Barrero J.M., Jacobsen J.V., Talbot M.J., White R.G., Swain S.M., Garvin D.F., Gubler F. (2012). Grain dormancy and light quality effects on germination in the model grass *Brachypodium distachyon*. New Phytol..

[36] Oracz K., Voegele A., Tarkowska D., Jacquemoud D., Turecková V., Urbanová T., Strnad M., Sliwinska E., Leubner-Metzger G. (2012). Myrigalone A inhibits *Lepidium sativum* seed germination by interference with gibberellin metabolism and apoplastic superoxide production required for embryo extension growth and endosperm rupture. Plant Cell Physiol..

[37] Railton I.D., Wareing P.F. (1973). Effects of abscisic on the levels of endogenous gibberellin-like substances in *Solanum andigena*. Planta.

[38] Kim Y.H., Choi K.I., Khan A.L., Waqas M., Lee I.J. (2016). Exogenous application of abscisic acid regulates endogenous gibberellins homeostasis and enhances of oriental melon (*Cucumis melo* var. L.) against low temperature. Sci. Hort..

